# *Mycobacterium haemophilum* and Lymphadenitis in Immunocompetent Children, Israel 

**DOI:** 10.3201/eid1409.070917

**Published:** 2008-09

**Authors:** Yishai Haimi Cohen, Jacob Amir, Shai Ashkenazi, Tal Eidlitz-Markus, Zmira Samra, Lea Kaufmann, Avraham Zeharia

**Affiliations:** Schneider Children’s Medical Center of Israel, Petah Tiqwa, Israel (Y. Haimi Cohen, J. Amir, S. Ashkenazi, T. Eidlitz-Markus, A. Zeharia); Rabin Medical Center, Petah Tiqwa (Z. Samra, L. Kaufmann); Tel Aviv University, Tel Aviv, Israel (Y. Haimi Cohen, J. Amir, S. Ashkenazi, T. Eidlitz-Markus, Z. Samra, A. Zeharia)

**Keywords:** Mycobacterium haemophilum, nontuberculous mycobacteria, lymphadenitis, children, Israel, dispatch

## Abstract

The database of a major microbiology laboratory in Israel was searched to determine the prevalence of nontuberculous mycobacterial lymphadenitis in immunocompetent children. We observed a 4-fold increase in nontuberculous mycobacteria isolates during 1985–2006, which was attributable mainly to increased detection of *Mycobacterium haemophilum* starting in 1996.

Nontuberculous mycobacteria (NTM) are a common cause of nonpyogenic craniofacial lymphadenitis in otherwise healthy children. *Mycobacterium avium* complex (MAC) is the main pathogen (*1*–*3*). *M*. *haemophilum* is traditionally considered a cause of NTM in immunocompromised patients (*4*–*7*), although a recent study from the Netherlands found that it is also common in immunocompetent children (*8*).

## The Study

Prompted by the increasing number of *M*. *haemophilum* isolates identified at our tertiary medical center in Israel in the past decade, we investigated the current prevalence and clinical characteristics of NTM lymphadenitis in immunocompetent children. The database of our microbiology laboratory was searched for all NTM-positive cervical lymph node cultures of children during 1985–2006. In addition, we reviewed records of the Day Hospitalization Unit (DHU) for all patients with a diagnosis of NTM lymphadenitis from January 1996 (when *M*. *haemophilum* was first isolated in our laboratory) through December 2006. Data obtained were patient age and sex, *Mycobacterium* species, ethnic background (Jewish/Arab), medical history, duration of node enlargement until referral, site affected, number of infected sites, size of nodes at initial visit (measured by the clinician), discoloration of the skin overlying the lymph nodes, and maximal induration in response to purified protein derivative (PPD). Patients with *M*. *haemophilum* infection were compared with those with MAC infection.

All specimens were processed for direct Ziehl-Neelsen staining. From 1985 through 1995, specimens were placed on solid Lowenstein-Jensen (L-J) medium. Thereafter, liquid MB Redox broth and liquid Bactec 460 12B medium (Becton Dickinson Microbiology Systems, Cockeysville, MD, USA) were added to the L-J medium. Toward the end of 1999, Bactec medium was replaced with the liquid Mycobacteria Growth Indicator Tube system (Becton Dickinson Microbiology Systems). A hemin-containing paper strip (X-factor) was regularly placed into the 2 liquid media, which were incubated with the L-J medium at 37°C and 30°C.

Discrete variables were compared between groups by using a Pearson χ^2^ test or Fisher exact test, as appropriate. Continuous variables were compared with 1-way analysis of variance. A p value <0.05 was considered statistically significant. Data were analyzed by using BMDP software (www.statsol.ie/html/bmdp/bmdp_home.html). The study was reviewed and approved by the local ethics committee.

The laboratory database contained 111 NTM isolates during 1985–2006, of which 77 (69%) were in samples from patients who visited the DHU from January 1996 through December 2006. Species distribution was as follows: MAC, 54; *M*. *haemophilum*, 41; others, 16. The Figure shows the increase in isolation rate of NTM and *M*. *haemophilum* since the initial isolation of *M*. *haemophilum* in March 1996.

**Figure:**
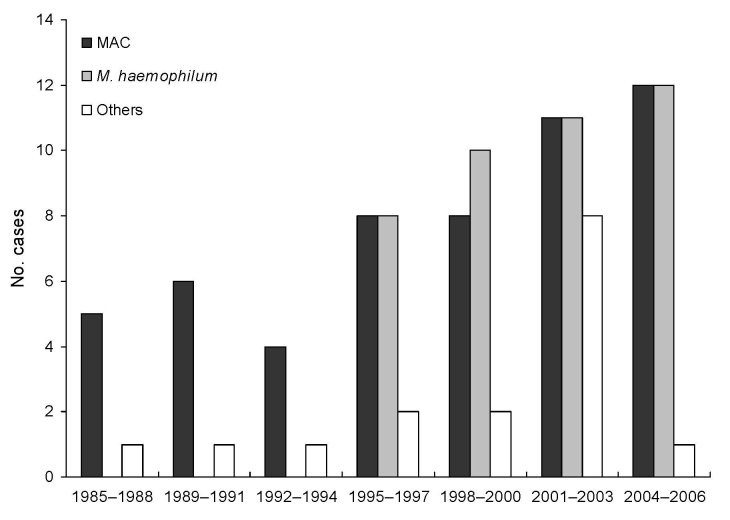
Distribution of nontuberculous mycobacteria species (*Mycobacterium avium complex* [MAC], *M*. *haemophilum*, and others) isolated from craniocervical lymph nodes of immunocompetent children in Israel, 1985–2006.

The 77 patients managed at our DHU for NTM lymphadenitis included 38 boys (49%) and 39 girls 8 months to 15.5 years of age (median 2.4 years). *M*. *haemophilum* was isolated from 39 children and MAC from 29. The demographic and clinical features of these children are shown in the Table. The patients in the *M*. *haemophilum* group were significantly older than those in the MAC group (mean 4.7 years vs. 2.3 years; p<0.001); 9 patients with *M*. *haemophilum* infection (23%) were >7 years of age compared with none with MAC infection. Mean time to referral was significantly longer in the *M*. *haemophilum* group (1.5 months vs. 1.1 months; p = 0.045). No statistically significant differences were noted for the other parameters studied.

**Table:** Demographic and clinical characteristics of children with *Mycobacterium haemophilum* and *M. avium* complex lymphadenitis*

Variable	*M. haemophilum*, n = 39	*M. avium* complex, n = 29	p value
Mean age, y (range)	4.7 (8 mo–15.5 y)	2.3 (9 mo–7y)	<0.001
Male/female, no. (%)	17/22 (44/56)	16/12 (57/43)	0.33
Ethnic origin, no. (%)			
Jewish	26 (81.2)	22 (81.5)	1.0
Arab	6 (18.8)	5 (18.5)	
Mean time to referral, mo (range)	1.47 (0.25–5)	1.1 (0.25–3)	0.045
Site, no. (%)			
Submandibular	24 (61.5)	13 (46.4)	0.32†
Neck	7 (17.9)	6 (21.4)	
Preauricular	3 (7.7)	3 (10.7)	
Occipital	–	1 (3.6)	
Cheek	–	4 (14.3)	
Bilateral submandibular	1 (2.6)	–	
Submandibular and preauricular	3 (7.7)	1 (3.6)	
Neck and preauricular	1 (2.6)	–	
No. infected sites: 1/2, no. (%)	34/5 (87/13)	27/1 (96/4)	0.72
Side: right/left, no. (%)	23/16 (59/41)	21/7 (76/24)	0.20
Mean size of lymphadenopathy, cm^2^ (range)	8.7 (1.0–25)	8.9 (1.0–35)	0.93
Skin discoloration: yes/no, no. (%)	15/14 (52/48)	9/9 (50/50)	1.0
PPD: mean maximal induration, cm (range)	17.9 (0–32)	14.5 (0–45)	0.22

*PPD, purified protein derivative. †Refers solely to the submandibular site.

## Conclusions

We speculate that the nearly 4-fold increase in the recovery rate of NTM from lymph nodes of immunocompetent children in the past 22 years at our center was attributable to the emergence of *M. haemophilum* as a major pathogen of craniocervical lymphadenitis starting in 1996. This assumption is supported by the only slight increase in the other NTM pathogens during the study period. The increased prevalence of NTM cervical lymphadenitis may be explained by conversion of our hospital to a tertiary pediatric center in 1991, concomitant with the growing awareness of NTM as a cause of craniocervical lymphadenitis among its physicians. Furthermore, the rate of isolation of *M*. *haemophilum* in this study was 51%. Another study from the Netherlands reported a similarly high prevalence rate (44%) of *M*. *haemophilum* lymphadenitis in immunocompetent children (*8*).

Although the distribution of NTM species may depend on local ecologic factors (*9*), given the wide geographic range of *M*. *haemophilum* infection in immunocompromised children (*4*–*7*) we would have expected to see reports of increased infection in immunocompetent children from >2 countries. We suspect that the change in our laboratory’s processing procedure in 1996 to include broth with an iron supplement and incubation of the samples at 30°C in addition to 37°C (*10*) contributed to the high isolation rate. Our failure to use these conditions before 1996 could have led to an underdiagnosis of *M*. *haemophilum* infection; this may also be true for other laboratories (*11*).

These findings suggest that a failure to isolate a pathogen in children with suspected mycocbacterial craniocervical lymphadenitis, especially those >7 years of age, should prompt a targeted laboratory search for *M*. *haemophilum* by using proper culture conditions or molecular techniques (*10*–*12*). Identification of NTM infection has serious clinical implications because it can spare these patients, who often have a positive PPD response and cytologic results compatible with tuberculosis, unwarranted, prolonged antituberculosis therapy. The mean PPD response in our patients (>14.5 mm) confirms the lack of value of PPD in distinguishing NTM infection from tuberculosis (*13*).

The higher mean age of the children with *M*. *haemophilum* infection in our series compared with that of children in the MAC group is consistent with findings of a study in the Netherlands (*8*). Our finding may be explained by the younger age at which children are exposed to playgrounds, which are presumably linked to MAC infection, than to swimming pools, which are presumably linked to *M*. *haemophilum* infection (*8*). However, the mean age of our patients was lower by >1 year than the age of the Dutch children (*8*), perhaps the result of warmer climate and of the younger age of daycare attendees in Israel, both of which are associated with longer and earlier exposure to sandpits and swimming pools.

In contrast to the results of the Dutch study (*8*), ethnicity was not a risk factor; the rates of affected Jewish and Arab children matched the distribution of these ethnicities in the general population of Israel. This finding may reflect the similar environmental conditions to which these ethnic groups are exposed.

Despite the wide variability in the interval from onset of lymph node swelling to patient referral for investigation, the mean time of ≈5 weeks is consistent with that in previous studies (*1*–*3*). The difference of ≈10 days between the 2 groups in our study, although statistically significant, was not of clinical importance. The longer interval in the *M*. *haemophilum* group might have been caused by less attention parents tend to pay to physical changes in older children than in infants. In contrast to the findings in the Dutch study (*8*), we noted no predilection to multisite infection in either group, and all 4 patients with extranodal (cheek) involvement were infected with MAC.

In conclusion, *M*. *haemophilum* is an emerging pathogen in nonpyogenic craniocervical lymphadenitis in immunocompetent children in Israel. *M*. *haemophilum* infection usually affects older children (>7 years of age) but is otherwise clinically similar to MAC infection.
